# A systematic review of the effects of nicotine replacement therapy on agitation among nicotine-dependent psychiatric inpatients

**DOI:** 10.1192/bjo.2021.785

**Published:** 2021-06-18

**Authors:** Joseph Toms, Jacob King

**Affiliations:** 1 Queen Elizabeth Hospital King's Lynn NHS Foundation Trust; 2 University College London Hospitals NHS Foundation Trust

## Abstract

**Aims:**

This systematic review aims to evaluate the effect of nicotine replacement therapies (NRTs) on measures of agitation amongst nicotine-dependent adult psychiatric inpatients.

**Background:**

Since the introduction of the smoke-free policy for all psychiatric facilities, a psychiatric admission is likely to upset a nicotine-dependent individual's normal routine of nicotine consumption. In addition to the physiological effects of nicotine withdrawal (NW), the interpersonal dynamic which nurse-led guardianship of nicotine products constructs presents stressors to the nicotine dependent patient.

Several systematic reviews evaluating changes in objective measures of agitation amongst smoking patients in medical critical care units have found varied results, with some demonstrating worsening agitation with NRT use. We therefore believe that there is sufficient equipoise in the use of NRT to prompt a review of studies amongst psychiatric inpatients.

**Method:**

This review identified English language studies through developed search strategies in PubMed/MEDLINE, EMBASE, PyschINFO, PSYCHLit, Cochrane databases, and Google scholar. The bibliographies of notable papers were explored. Hand searches of five major psychiatric journals were conducted. Peer reviewed studies of any study design were included if they reported primary data of adult psychiatric inpatients. Studies were extracted from 1990 – present, this was felt appropriate as nicotine replacement patches became available in 1992.

Search strategies were informed by MeSH search terms and included multiple conceptions of “agitation”, including variations on; agitation, irritability, and arousal to capture the concept from broad academic constructions. The quality of studies was assessed with the Newcastle-Ottawa and Cochrane Collaboration tools.

This review follows PRISMA guidelines, and an application for PROSPERO registration has been submitted pending acceptance.

**Result:**

Two studies were identified which matched inclusion criteria. A double-blinded randomised placebo-controlled trial of 40 nicotine-dependent inpatients from Allen et al. reported a significant 23% reduction in Agitated Behaviour Scores at 24 hours following NRT administration on admission compared to their matched placebo controls. Yet a retrospective cross-sectional analysis from Okoli using scores for NW identified more severe withdrawal symptoms including “restlessness” and “anger/irritability” than nicotine-dependent patients not provided with NRT.

**Conclusion:**

Despite considerable commentary within literature there is presently only one study providing moderate evidence of a positive benefit to measures of agitated behaviour from the use of NRT amongst nicotine-dependent psychiatric inpatients. There is currently very low evidence whether NRT improves or exacerbates the agitation associated with NW amongst nicotine-dependent psychiatric inpatients.

